# Feasibility of the Positive Thoughts and Actions Prevention Program for Middle Schoolers at Risk for Depression

**DOI:** 10.1155/2011/241386

**Published:** 2010-09-05

**Authors:** Carolyn A. McCarty, Heather D. Violette, Elizabeth McCauley

**Affiliations:** ^1^Seattle Children's Hospital and University of Washington, Center for Child Health, Behavior, and Development, 1100 Olive Way, Suite 500, MS MPW 8-1 Seattle, WA 98101, USA; ^2^Seattle Children's Hospital, 4800 Sand Point Way NE, MS W-3636, Seattle, WA 98105, USA

## Abstract

Despite the importance of adolescent depression, few school-based prevention programs have been developed and tested in the United States with middle school populations. This study examined the acceptability and changes in targeted outcomes for a new preventative program, Positive Thoughts and Actions (PTA). Sixty-seven 7th grade students with elevated depressive symptoms were recruited from public schools and randomized to the 12-week PTA program with a parent-component or to a school-as-usual control group. The PTA prevention program was well received by students and parents, yielding high rates of participation and satisfaction among those randomized to receive the intervention. However, analyses of the efficacy of the program in changing depressive symptoms were not significant. In terms of our proximal program targets, most differences were not statistically significant, though effect sizes suggested advantage of PTA over control group in coping, cognitive style, and parent-child communication. This preliminary research highlights a need for further testing of programs for school-based prevention of depression and promotion of positive emotional health.

## 1. Introduction

Elevated levels of depressive symptoms can be detrimental for adolescents because they may interfere with important developmental processes and lead to a cascade of adjustment difficulties [1]. Youth with depressive symptoms are at significant risk for meeting diagnostic criteria for a depressive disorder later in adolescence [2] and in adulthood [3]. Depression and depressive symptoms are a primary risk for suicide, a leading cause of death for adolescents [4]. Longitudinal research has shown substantial continuity of youth depression into adulthood, with impaired functioning in work, social, and family life, and elevated risk of adult suicide attempts and completed suicide [5, 6]. A recent report by the National Research Council and the Institute of Medicine concludes that it is critical to shift the focus to advancing health and preventing disorders from occurring in the first place, rather than waiting until a disorder is well established and has done considerable harm [7]. The goal of the current paper is to describe the development of a prevention model that addresses the needs of middle/junior high school students at risk for depression.

Schools play an increasingly important role in providing mental health services to children [8]. For the majority of children and adolescents, the school system provides the only source of mental health service [9]. Despite the importance of addressing depression and mental health as an overall component of youth health, only a handful of preventative programs targeting depression have been developed for middle schools, most of which have been tested in Australia [10–12]. In this country, the Penn Resiliency Program and the Coping and Support Training Program are the only two preventative interventions targeting depression that have been specifically developed for and empirically tested in middle/junior high schools [13, 14]. Thus overall, there is still a dearth of available programs targeted to meet the particular developmental needs of adolescents in this formative transition.

In response to a scarcity of curricula to address middle school stress and depression, the Positive Thoughts and Actions (PTA) program was developed and tested for feasibility in the current pilot study. The PTA program falls under the spectrum of mental health interventions as an indicated school-based prevention, while schools that operate under a positive behavioral support framework would classify the PTA program as a selected/targeted intervention [15]. The middle/junior high school timeframe was chosen because it marks a time of change and transition when youth adapt to numerous psychological, physical, cognitive, and social changes that are associated with an increase in psychopathology [16–18]. Our PTA curriculum was designed to address depressive symptoms through intervention on three proximal intervention targets. These intervention targets—coping, cognitive style, and parent-child communication—were chosen as indicators of outcome based upon their theoretical links to risk for depression. Addressing some of the risk factors that contribute to the escalation of depressive symptoms at this age may be important in preventing long-term adjustment difficulties that can arise from subclinical depressive symptoms. This developmentally based prevention program is unique and innovative in addressing key factors that contribute to and perpetuate depressive symptoms during the early adolescent years. 

First, evidence suggests that both youth and adults with depression have less adaptive and more limited coping repertoires compared to youth without depression [19, 20]. For example, youth with depression may use less primary control (efforts to cope by making objective conditions conform to one's wishes) and secondary control (efforts to cope by adjusting oneself to fit objective conditions). Second, certain cognitive styles, including excessive negative thoughts and low levels of perceived control are characteristics unique to the development of depression [21, 22]. Third, poor parent-child relationships and family communication difficulties serve as risk factors for the development of depression among youth [23]. 

Most tested intervention programs for adolescent depression, including those that are not school-based, have been delivered exclusively to the youth, without any parental involvement [23]. There are a number of reasons why the inclusion of parents in the intervention process may be particularly important for younger adolescents, including that reactions to difficult events or circumstances within the family can precipitate depressive symptoms, youth cannot change many aspects of their environment, and interventions can be more effective when they are implemented consistently across situations and persons. Providing psychoeducation to parents of depressed youth has been found to be beneficial by improving parents' coping skills and the family climate [24]. Moreover, several studies suggest that adolescents learn cognitive and coping styles from their parents and caregivers [25]. Thus far, only two other school-based depression prevention programs published in the literature have included an active parent component [12, 26]. 

This pilot trail of the PTA program was conducted to determine if the contextual focus of the PTA program was (1) acceptable to children and families, as indicated by their participation and satisfaction with intervention components, and (2) associated with improvements in youth's depressive symptoms, coping, cognitive style, and parent-child communication.

## 2. Methods

### 2.1. Subjects

A total of 67 7th grade students were recruited from 4 Seattle Public Middle schools after school wide screening for depression was conducted on a larger sample (*n* = 684) in Fall of 2005 and Fall of 2006. Students who scored higher than 14 (top 25%) on the Mood and Feelings Questionnaire (described below) after screening were invited to participate in the study. Exclusion criteria for students included (1) clinically elevated externalizing problems, (2) the presence of suicidal ideation, (3) probable diagnoses of Major Depressive Episode on the Patient Health Questionnaire—Adolescent Form, (4) plans to move to a nonparticipating school, and (5) parents who did not speak English. The first three exclusion categories were designed to ensure we were identifying youth who were appropriate for prevention and were not showing clinical levels of depression and related problem-behavior. Demographic characteristics of participating students and their families are provided in Table 1. We were able to retain 58 of the original 67 students (86.5%) for all followup assessments, as well as 60 of their parents (89.5%). 

### 2.2. Instruments

#### 2.2.1. Mood and Feelings Questionnaire (MFQ)

The MFQ was designed for children ranging in age from 8 to 18 and was written in parallel versions for parent and child, which both were administered in this study. The MFQ comprises both the full range of items assessing the DSM diagnostic criteria for depressive disorders as well as additional items reflecting common affective, cognitive, and vegetative aspects of childhood depression [27]. It has shown both high content validity and criterion validity [28]. Internal consistency (Cronbach's Alpha) was reported at  .90 in both parent and child samples. Parent-report items predict psychiatric versus pediatric patient status and depressed versus nondepressed status in clinical groups. The MFQ correlates highly with depression diagnoses and the Child Depression Inventory [29]. For this study, internal consistency (Cronbach's alpha) ranged from  .89 to  .94 across the four time points. 

#### 2.2.2. Children's Depression Rating Scale-Revised (CDRS)

The CDRS-R was administered to youth to assess the severity of depressive symptomatology. The CDRS-R is a clinician-rated scale used as a screening and diagnostic tool, consisting of 17 items scored from 1 to 5 or 1 to 7 [30]. The total score of the CDRS-R has been shown to be sensitive to change in severity of symptoms in treatment studies [31]. For this study, internal consistency (Cronbach alpha) ranged from  .71 to  .84 across the four time points. 

#### 2.2.3. Responses to Stress Questionnaire (RSQ)

The RSQ [32] measures a range of responses to stress, including voluntary or controlled coping responses and involuntary or automatic reactions. Students are asked to rate how much they used specific coping techniques when faced with specific recent stressors. Scores from two scales were used for the current study: (1) *primary control engagement coping (PCEC)*, encompassing problem solving, emotion regulation, and emotional expression, and (2) *secondary control engagement coping (SCEC)*, encompassing positive thinking, cognitive restructuring, acceptance, and distraction. Convergent and discriminant validity of the RSQ has previously been established [33]. In the current sample, internal consistency (Cronbach alpha) for primary control coping ranged from  .79 to  .86, and secondary control coping ranged from  .68 to  .84. 

#### 2.2.4. Personal Control Scale

The personal control scale is a 5-item scale assessing the degree to which the youth feels a sense of control over their mood, problems, and life in general. It has previously shown good internal consistency (*α* =  .77) among high-school students [34]. For this study, internal consistency (Cronbach alpha) ranged from  .82 to  .85.

#### 2.2.5. Children's Automatic Thoughts Scale (CATS)

CATS measures the frequency of negative thoughts, and has been validated on children aged 7–16 [35]. A 5-point rating scale ranging from 0 “not at all” to 4 “all the time” is used to rate 40 different automatic negative thoughts, including thoughts related to physical threat, social threat, personal failure, and hostility. Internal consistency of the subscales is high, with test-retest reliability adequate. In prior research, the CATS measure clearly discriminated clinically depressed youth from those with anxious and oppositional problems [35]. Internal consistency (Cronbach alpha) for this study was high, ranging from  .91 to  .96.

#### 2.2.6. Parent-Child Communication Scale (PCC)

The PCC Scale includes both parent and child-report forms, and was adapted from the Revised Parent-Adolescent Communication Form of the Pittsburgh Youth Study [36, 37]. The child measure assesses children's perceptions of their primary caregiver's openness to communication (10 items), and the parent measure (20 items) assesses both parent and child communication skills. Other studies have reported alpha coefficients for communication subscales ranging from  .66 to  .81 in 7th grade samples [38]. For this study, internal consistency (Cronbach alpha) for parent ratings of communication ranged from  .51 to  .81 across the four time points, whereas child ratings of communication ranged from  .76 to  .84. 

### 2.3. Procedures

All students who scored 14 or above on the MFQ were individually evaluated for clinical needs using a brief clinical evaluation protocol. Their parent or guardian was called and provided with feedback about the child's needs and referrals for resources, if indicated. Students and parents who met inclusion criteria and consented to participate were randomly assigned to the intervention group, Positive Thoughts and Actions (PTA) or the control group. 

PTA took place at school, consisting of 12 weekly (once per week) group-administered sessions, two home visits with parents and student together, and two group-based parent workshops, conducted in the evenings at the students' school. The PTA program included aspects of behavioral, cognitive, interpersonal, and family-systems interventions, the content of which is detailed in Table 2. PTA taught three major skills: thinking positively, taking positive action, and problem solving. Students applied these skills to self-identified problems/goals, and parents were given communication and problem-solving tools to help support their children. 

The control group participants received usual care in the school, meaning they were free to seek school-based (e.g., counseling) or other services (e.g., community mental health), but they were not provided with systematic intervention. Control group students attended their regular academic classes during the PTA student group time. Thirty-one students were assigned to the control group and 36 to the PTA intervention group. 

Trained graduate level interviewers conducted structured research interviews in the family home. All instruments were administered to students and parents by separate interviewers after explaining the instructions and answering their questions. Participants were interviewed using all study measures at four time points: Winter of 7th grade, prior to the start of intervention (Baseline), Spring of 7th grade, in the weeks following intervention (Postintervention), Winter of 8th grade, (6-months followup), and Fall/Winter of 9th grade, (18-months followup).

### 2.4. Data Analysis

Descriptive statistics were used to summarize demographic data. To determine the effects of the intervention, general linear model (GLM) repeated-measures analyses were conducted for each dependent variable with group (PTA versus control group) as the between-subjects variable and time as the within-subject variable. All analyses were conducted controlling for baseline levels depressive symptoms (CDRS). When significant time or group effects were found, posthoc contrasts were analyzed to determine the source of the individual differences. The statistical package used to run all analyses was SPSS (version 17.0), with statistical significance set at *P* <  .05. Effect sizes (ESs) were also computed for all variables in order to examine the magnitude and direction of effects, using the procedures for Cohen's d with adjusted means (difference between the adjusted means of the treatment and control group, divided by a pooled standard deviation) [39]. All ESs were calculated such that positive values implied an advantage for intervention over control group. 

## 3. Results

### 3.1. Participation and Satisfaction

Of the students randomized to the intervention group, 35 of 36 completed the prevention program, with an average attendance rate of 11 of 12 sessions for completers. One hundred percent of the parents of PTA youth received at least some of the parent intervention, and 94% received at least three of the four sessions. Twenty-six of the parent participants were mothers only (72%), 6 were fathers only (17%), 3 families had both parents participate (8%), and 1 “other caregiver” was the primary respondent (3%).

Parent satisfaction ratings were obtained following the initial parent-child home visit and the parent workshops. Of the 36 PTA parents, 72% of the parents found the initial home visit to be “very helpful”, 25% found it to be “somewhat helpful”, while one parent found the session to be “a little helpful”. Overall, the parent workshop components were rated to be “very helpful” (45%) or “somewhat helpful” (55%) by those who participated. 

Student satisfaction with their group membership and feelings about PTA class were gathered at week 11 of the intervention. Of the 36 PTA students, 48% liked the group they were in “very much”, 36% liked the group “pretty much”, and 13% felt the group was “all right”. One student (3%) disliked the group a little and felt “embarrassed” about being in the class. Twenty percent reported feeling neutral about being in the class. The remaining students felt “comfortable” (30%) or “very comfortable” (47%) about being in the class.

### 3.2. Depressive Outcomes

Total scores on the Children's Depression Rating Scale-Revised showed a significant main effect across time *F*_2,53_ = 4.89, *P* =  .01. Followup analyses revealed CDRS-R scores varied for the control group only (*F*_2,20_ =  8.67, *P* =  .002) with higher CDRS-R scores at post-intervention and 18-month followup compared to 6-month followup. No significant interaction effect between time *x* group (*F*_2,53_ =  .73, *P* =  .49) was found. Overall, parents reported fewer depressive symptoms than youth, as shown in Table 4. Parent ratings of depressive symptoms (MFQP) varied significantly across time, *F*_3,49_ = 4.10, *P* =  .01. Parents in the control group reported significantly fewer depressive symptoms at 6-month and 18-month followup than baseline, *F*_3,18_ = 3.62, *P* =  .03. For parents in the intervention group, ratings of their child's depression were lower at 6-month followup compared to baseline (*P* =  .03), though overall mean differences were nonsignificant, *F*_3,28_ = 1.71, *P* =  .19. No significant effects were found for depressive symptoms on the child-report MFQ (*P* =  .95). 

Examination of effect sizes for depression outcomes yielded a mixed picture, depending on time and informant (see Table 5). Youth PTA participants reported slightly higher mean levels of depressive symptoms after the intervention, compared to control youth, as indicated by the negative effect size value (ES = −.16). By 18 month followup, when participants were in the 9th grade, the pattern of results reversed, showing a slight advantage for PTA participants (ES =  .18). Effect sizes based upon parental report suggested that parents of youth in the intervention group reported more depressive symptoms among their children at postintervention and 18-month followup. The CDRS yielded neutral to medium *negative* effect size values, although the two groups were not well matched in their CDRS scores at baseline. 

### 3.3. Coping, Cognitive Style, and Parent-Child Communication

As for the three proximal intervention targets, primary control coping showed a significant main effect for group, *F*_1,53_ = 7.22, *P* =  .01. PTA participants were found to have significantly higher mean levels of primary control engagement coping than the control group. Follow-up repeated-measures for group showed the PTA participants demonstrated significant improvements in coping at post-intervention compared to baseline (*F*_3,29_ = 7.43, *P* =  .01). No significant interaction effect between time x group for primary coping (PCEC) were found (*P* =  .86), nor were differences in secondary coping (SCEC) on the RSQ significant (*P* =  .20). In terms of our two measures of cognition, differences across time and group were found for one of these outcomes. Personal control scale showed a significant time x group interaction, *F*_3,52_ = 3.61, *P* =  .02. Followup analyses revealed differences for both groups, with mean differences shown in Table 3. PTA participants had significantly higher mean levels of perceived control at 18-month follow-up compared to baseline (*F*_3,30_ = 2.92, *P* =  .05), while control group youth had significantly higher mean levels at 6-month follow up compared to post-intervention (*F*_3,19_ = 3.70, *P* =  .03). No significant effects between groups were found for automatic negative thoughts (*P* =  .92), though effect sizes suggested small advantages for the treatment group over all time points (see Table 4). 

Finally, for parent-child communication, no significant differences were found for communication on the PCC parent or child versions, *P* =  .61 and *P* =  .31, respectively. Effect sizes suggested that both parent and youth participants in the intervention condition reported better parent-child communication following intervention, but that these improvements relative to the control group did not sustain over time. 

## 4. Discussion

The Positive Thoughts and Actions prevention program was well received by students and parents, yielding high rates of participation and satisfaction among those randomized to receive the intervention. Nearly 84% of adolescent participants reported liking the group. While indicated prevention programs that target individuals who display some early signs or symptoms have been criticized for the potential for increased labeling and stigma, we found that students' perceived embarrassment as a result of participation was low, with 77% reporting feeling “comfortable” or “very comfortable” participating, and another 20% reporting “neutral” feelings. These low levels of stigma are consistent with those reported among participants another school-based depression prevention, the Adolescents Coping with Emotions program [40]. Overall, the program was acceptable to students and families, and the structure of the intervention was conducive to participation. The conceptual framework for the program, intervention targets, and inclusion of developmentally salient applications, such as learning, relationships, and making healthy decisions, were well received by our partner schools.

Analyses of the efficacy of the PTA program relative to the control group in changing depressive symptoms were not significant. Effect-size patterns were inconsistent across time and informant, with some negative effect-size values which could indicate iatrogenic effects of the PTA group. However, given the degree of scatter in mean values for youth-report measures, the fact that the PTA participants had higher CDRS scores at baseline, and the decrease in parent-reported depressive symptoms across time for both groups, it is difficult to discern the overall impact of PTA with these data. Moreover, effect size estimates using small sample sizes are prone to bias [41], and this can be further exacerbated when measuring episodic phenomena such as depressive symptoms. The PTA program developers are currently revising program materials based on input from consumers and two depression prevention expert consultants, and will conduct a larger trial as a next step. Increased sample size and power will allow for stronger conclusions about intervention effects to be made, but does not replace the importance of publishing these pilot data [42].

In terms of our proximal program targets, effect sizes suggested an advantage of the PTA over control group in each area, though most differences (aside from primary control engagement coping) were not large enough to achieve statistical significance. The largest and most robust increases for PTA participants were in areas of personal control and primary control engagement coping over time. Personal control and coping are common targets of preventative programs targeted to youth with internalizing problems, such as depression or suicidal ideation [34]. Gains in self-efficacy and control are associated with decreased vulnerability to suicide [43, 44] and moreover these skills are potentially important in and of themselves because they confer a sense of autonomy and increased personal resources for handling stress. Knowledge of coping skills can reduce and prevent negative consequences of stress in adolescence, thereby having far-reaching benefits that extend beyond a specific disorder. Resiliency research has linked positive adjustment to successful coping with developmental challenges. In terms of magnitude, differences in primary control engagement coping between PTA and control groups were “medium” in size. 

However, it is important to note that despite these changes in primary control engagement coping, none of the changes in depressive symptoms were significant over time for PTA participants, showing a disconnect between outcomes. While we had conceptualized coping, cognitive style, and parent-child communication to be potential mechanisms for change in depressive symptoms, our results were not consistent with a meditational model because mediation is predicated on observing a clear intervention effect. Few other studies have examined mechanisms of change in depression intervention [45], and those that have tested for mediation have not found evidence that cognition necessarily mediates outcome in CBT outcome [46]. 

PTA stands out as one of the first programs to successfully implement a school-based intervention focused on depression with high levels of parent engagement. We were able to successfully engage at least one parent from all families through our outreach efforts to meet with them at their home or another convenient location. Meetings were scheduled in the evenings or on the weekends, at a time that worked best for the family. The same intervention specialist who would be leading student groups set up a meeting with the family prior to the first group. Surveys of parent availability were conducted prior to scheduling all parent workshops to maximize attendance. Intervention specialists made personalized reminder calls prior to each workshop and family meeting to engage parents. In the final student group, students practiced giving a short speech about what they gained from the experience. Then, in the final family session, students presented this synopsis to their parent(s), as well as demonstrating some of the concepts learned from the PTA curriculum. All in all, the close tie between student groups and the parent component was believed to help foster parents' engagement and interest in participating.

This participation rate is higher than that found by other depression prevention programs based in schools. For example, in the Australian test of the resourceful adolescent program (RAP), only 36% of adolescents had a parent attend any session and 10% attended all 3 sessions. Our rates of parent participation in any intervention were similar to those achieved by the Penn Resiliency Program (PRP), where parents of 91% of students attended at least one of the parent intervention component sessions. However, our study yielded a much larger uptake when considering the percentage of parents participating in the majority of the intervention, with 94% of our parents completing at least three of the four parent sessions. The PRP parent intervention consisted of six 90-minute sessions targeted to parents' cognitions and coping skills, with 41% of parents attending at least five of the six sessions [26]. Thus, the results of our study, conducted in urban Seattle, and those of the PRP, conducted in suburban Pennsylvania, together demonstrate that it is possible to achieve high rates of parental involvement for school-based prevention programs when adequate resources for outreach are provided, such as meeting with parents at their home and at their convenience. 

## 5. Conclusion

Schools are faced with increasing pressure to address the emotional wellbeing of students to promote learning and healthy development. Building programs to address mental health is a critical component to improving overall school health. This preliminary research highlights the feasibility of school-based prevention programs addressing depression and promotion of positive emotional health. The most significant limitation of this research is the small sample size that was used in this feasibility trial. While it is difficult to draw conclusions about the outcome of the intervention given the small sample size, the intervention was well received, and parents were particularly engaged. However, it possible that identification and attention alone contributed to parent and student satisfaction, and both conditions were provided with a brief clinical evaluation and feedback, which may have had some effect on outcomes. Study information contributes to the meager understanding of the feasibility and uptake of school-based preventative interventions addressing depressive symptoms during middle/junior high school, and lays the foundation for further research examining the effectiveness and value of such programs for consumers, including students, parents, and school personnel. Future examination of the PTA program in additional schools with a larger sample will determine the effectiveness as a depression prevention curriculum. 

## Figures and Tables

**Table 1:** Demographic characteristics of participants.

Characteristics	PTA group *n* = 36		Control group *n* = 31	
Mean Age (SD)	12.97	(0.36)	13	(.40)
Sex, *n* (%)				
Female	20	(55.6)	14	(45.2)
Male	16	(44.4)	17	(54.8)
Race, *n* (%)				
White	24	(66.7)	19	(61.3)
African American	1	(2.8)	3	(9.7)
Asian	2	(5.6)	3	(9.7)
Native American	2	(5.6)	—	—
Other	7	(19.4)	6	(19.4)
Ethnicity, *n* (%)				
Hispanic	1	(2.8)	6	(19.4)
Non-Hispanic	35	(97.2)	25	(80.6)
Parental Education, *n* (%)				
HS Diploma/GED/Some College	13	(36)	12	(39)
Associates/Bachelor's Degree	18	(50)	15	(48)
Masters/Professional/Doctoral Degree	5	(14)	4	(13)
Family Constellation, *n* (%)				
Single (1 parent family)	15	(42)	9	(29)
Married (or 2 cohabitating parent)	21	(58)	22	(71)

**Table:** (a)

Student content		

Session no.	Title	Content
Week 1	Introduction and Purpose	To convey the purpose of the program, build a positive peer group, and to practice being positive with others
Week 2	Setting Goals	To motivate students to identify areas of their lives they would like to change and to select targeted goals and specific steps to take within each program area
Week 3	Start with Action	To teach the importance of getting active to improve mood and persist in goals
Week 4	Positive Thoughts, Positive Feelings	To understand the link between thoughts and actions and to increase positive thinking
Week 5	Changing the Way We Think and Feel	To identify negative and irrational thoughts and change them to be more positive and realistic
Week 6	STOP before Responding	To help students recognize when they are having an emotional reaction and to regulate their affect
Week 7	Making Decisions & Problem-Solving	To teach a 5-step approach to making decisions and solving problems
Week 8	Managing Conflict & Anger	To help students manage moods by controlling anger and resolving conflicts more productively
Week 9	Learning	To apply the skills learned to identified school goals
Week 10	Relationships	To practice applying Positive Thoughts and Actions to relationships
Week 11	Making Healthy Decisions	To adopt more healthy behaviors
Week 12	Staying on Track & Celebration	To recognize progress and identify areas of continued effort

**Table:** (b)

Parent content		

Session type/no.	Title	Content
Home Visit no. 1	Getting to Know Each Other	To build rapport, to assess strengths and needs, and to help clarify the parents' supportive role
Parent Workshop no. 1	Positive Thoughts & Actions for Parents	To teach parents perspective-taking, and to provide an overview to the parent of emotion regulation strategies
Parent Workshop no. 2	Communicating with Your Teen	To give parents an opportunity to learn and practice different ways to communicate about feelings and/or problems
Home Visit no. 2	Staying Successful	To provide an opportunity for the student to summarize key concepts and identify how the parent can support them

**Table 3:** Adjusted mean scores on depressive measures by group and time.

Construct	Depressive Symptoms		
Measure	MFQ-C	MFQ-P	CDRS
PTA			
Baseline	14.42 (9.85)	10.51 (10.17)	26.17 (7.50)
Post-intervention	15.91 (10.24)	9.11 (11.27)	27.45 (7.43)
6-month followup	10.86 (10.59)	7.37 (7.64)	25.67 (7.77)
18-month followup	16.17 (10.83)	9.28 (8.42)	27.75 (9.04)
Controls			
Baseline	14.87 (10.41)	10.67 (7.22)	23.95 (6.17)
Post-intervention	14.50 (7.41)	8.05 (6.44)	27.30 (6.78)
6-month followup	11.67 (6.83)	7.57 (5.65)	22.96 (4.01)
18-month followup	18.10 (10.96)	6.01 (5.26)	27.01 (9.61)

Standard deviations are in parentheses. MFQ-C: Mood and Feelings Questionnaire-Child; MFQ-P: Mood and Feelings Questionnaire-Parent; CDRS: Children's Depression Rating Scale.

**Table 4:** Effect sizes (Cohen's D) for outcomes across time.

Construct	Coping		Cognition		Parent-child communication		Depressive symptoms		
Measure	PCEC	SCEC	PC	CATS	PCCC	PCCP	MFQ-C	MFQ-P	CDRS
Postintervention									
	.67	.51	.64	.17	.35	.39	−.16	−.12	−.02
6-month followup									
	.52	−.05	−.32	.2	−.04	.15	.09	.03	−.44
18-month followup									
	.5	.16	.2	.24	.04	−.01	.18	−.47	−.08

Effect sizes were calculated using adjusted means from GLM (reported in Table 3).

(+) = Treatment > Control (better than); (−) = Treatment < Control (worse than).

**Table 5:** Adjusted mean scores on proximal outcome measures by group and time.

Construct	Coping		Cognition		Parent-Child Communication	
Measure	PCEC	SCEC	PC	CATS	PCCC	PCCP
PTA						
Baseline	25.88 (4.37)	30.93 (4.97)	20.78 (5.65)	25.06 (18.75)	19.93 (3.15)	25.56 (3.28)
Postintervention	26.15 (5.74)	32.42 (7.54)	21.79 (5.39)	19.92 (14.84)	19.29 (4.61)	25.64 (2.86)
6-month followup	26.04 (5.14)	21.61 (6.03)	21.61 (6.03)	19.61 (19.55)	19.60 (2.77)	25.24 (2.90)
18-month followup	26.10 (6.17)	22.93 (5.22)	22.93 (5.22)	19.67 (19.84)	19.31 (3.93)	24.58 (3.73)
Controls						
Baseline	22.81 (5.17)	30.06 (6.43)	19.63 (6.13)	26.15 (20.68)	19.76 (4.07)	24.93 (3.61)
Post-intervention	22.48 (5.23)	28.85 (6.33)	18.27 (5.59)	22.55 (15.92)	17.83 (3.56)	24.51 (2.88)
6-month followup	23.42 (5.03)	23.31 (4.48)	23.31 (4.48)	23.77 (22.06)	19.72 (3.35)	24.80 (3.04)
18-month followup	23.61 (3.28)	21.89 (5.08)	21.89 (5.08)	24.48 (19.06)	19.15 (4.24)	24.62 (3.75)

Standard deviations are in parentheses. PCEC: Primary Control Engagement Coping; SCEC: Secondary Control Engagement Coping; PC: Personal Control; CATS: Children's Automatic Thoughts Questionnaire; PCCC: Parent Child Communication—Child; PCCP: Parent Child Communication—Parent.
